# Does Cognitive Disengagement Syndrome Affect the Cognitive Flexibility of Children with Attention Deficit Hyperactivity Disorder?

**DOI:** 10.5152/eurasianjmed.2025.251160

**Published:** 2025-10-23

**Authors:** İbrahim Adak, Esin Özdeniz Varan, Özalp Ekinci, Ayşim Alpman, Zeynep Durmuş, Nergis Eyüpoğlu, Oğuz Bilal Karakuş, İpek Süzer Gamlı

**Affiliations:** 1 Department of Child and Adolescent Psychiatry, Erenköy Mental and Neurological Diseases Training and Research Hospital, University of Health Sciences, İstanbul, Türkiye; 2 Department of Clinical Psychology, University of Health Sciences, İstanbul, Türkiye

**Keywords:** Attention deficit disorder with hyperactivity, child and adolescent psychiatry, cognitive flexibility, neuropsychological tests, sluggish cognitive tempo

## Abstract

**Background:**

Cognitive flexibility (CF) is an ability to adapt to a changing environment, which is a prominent skill in children at school age. ADHD is a common disorder of childhood and can be accompanied by cognitive disengagement syndrome (CDS, previously referred to as “sluggish cognitive tempo”). This study aimed to assess CDS’s effect on CF in children with ADHD by using neuropsychological tests.

**Methods:**

The study sample consisted of 100 ADHD children aged between 6 and 12 years, including 2 groups: 60 ADHD-only and 40 CDS+ADHD. ADHD diagnosis and CDS symptoms in participants were assessed by Diagnostic and Statistical Manual of Mental Disorder Fifth Edition Text Revision (DSM-5-TR) based psychiatric interviews and rating scales. The Neuropsychological Battery, consisting of 4 different tests, Wisconsin Card Sorting Test (WCST), Stroop Color–Word Test, Verbal Fluency Test (VFT), and Color Trail Test, was applied to participants to compare CF of the ADHD-only group to CDS+ADHD.

**Results:**

It was found that the CDS+ADHD group showed lower performance than the ADHD-only group in the WCST and the Semantic Fluency Test—a subtest of the VFT. However, no significant performance differences were found between the groups in other tests.

**Conclusion:**

It was revealed that CDS co-occurrence causes lower CF performance in ADHD-diagnosed children. A more comprehensive approach is required to understand the nature of this difficulty.

**Main Points**

- Cognitive disengagement syndrome (CDS) commonly accompanies ADHD cases.
- Cognitive flexibility (CF) is a core executive function and essential for academic achievement, especially during childhood.
- Research on the impact of CDS on CF is still limited.
- This study found that CDS co-occurrence causes CF dysfunctions in children with ADHD, highlighting the need for more comprehensive assessment and tailored interventions.

## Introduction

ADHD is characterized by deficits in attention and/or impulsivity-hyperactivity. It is a neurodevelopmental condition that affects about 8% of children and adolescents worldwide.^1^ It is one of the most prevalent mental health diagnoses in child and adolescent psychiatry.^2^ The increasing number of newly diagnosed cases and the consistency of symptoms create major concern for public health.^3^

ADHD is classified into 3 subtypes based on the presence of different symptoms.^4^Following the introduction of subtyping in the Diagnostic and Statistical Manual of Mental Disorder Third Edition (DSM-III), a subset of individuals exhibiting attentional difficulties along with hypoactivity, mental confusion, and daydreaming was identified.^5^ This constellation of symptoms was termed sluggish cognitive tempo (SCT), which has subsequently garnered substantial research interest and has been validated as a distinct phenomenon from ADHD.^6^ In 2023, the term SCT was officially renamed cognitive disengagement syndrome (CDS) by a working group.^7^ This transition is undoubtedly a result of growing literature indicating that the previously identified core deficits, slowed response times, and diminished cognitive processing speed do not account for the syndrome.^8^ Contemporary research suggests that variations in cognitive orientation, specifically attention decoupling or disengagement, may underlie the syndrome.^9^ This shift in understanding underlines the need for further investigation into these cognitive mechanisms. Epidemiological data indicate that 25%-40% of young individuals with ADHD exhibit comorbid CDS.^10^^,^^11^ It is also well-documented that youths with isolated CDS typically do not present to child and adolescent psychiatry clinics unless they have concurrent ADHD.^12^

Cognitive flexibility (CF), a critical component of executive function, is an ability to adjust attention and cognitive processes in response to changing external stimuli.^13^ It is found to be related to higher scholastic achievement in mathematics and reading skills in school-aged children.^14^^,^^15^ Cognitive flexibility can be assessed through self-report measures, neuropsychological tests (NPTs), or neuroimaging, each with its inherent limitations.^16^

Consequently, this study aims to compare CF skills in children with ADHD-only versus CDS+ADHD. This research seeks to advance the understanding of CDS and potentially contribute to the development of novel diagnostic and therapeutic strategies by examining how attentional disengagement influences CF in children with CDS.

## Material and Methods

### Participants

**Figure 1.**
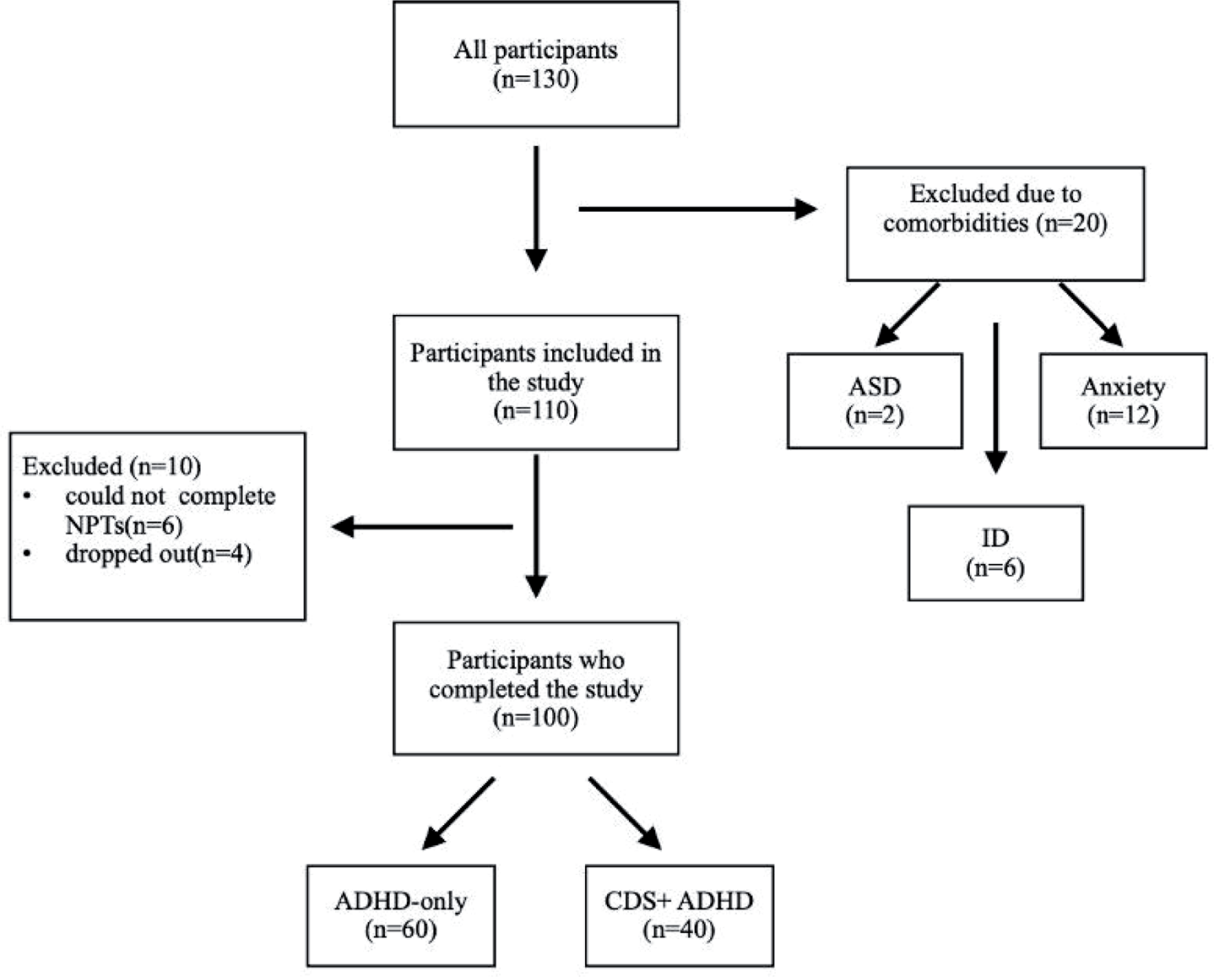
Flowchart of the current study.

Participants were recruited from children who applied to Erenköy Mental and Neurological Diseases Training and Research Hospital in Istanbul from April to September 2024. The inclusion criteria were determined as follows: 1) Being between the ages of 6 and 12; 2) Being diagnosed with ADHD according to DSM-5 TR; 3) Not having epilepsy and hearing/visual impairment; 4) Not having any psychiatric comorbid disorder. 130 participants consisting of both newly and previously ADHD-diagnosed children were recruited. Of these, 20 were excluded due to the presence of psychiatric comorbid disorders and 10 of them were excluded for not completing NPT (6) or dropping out of the study (4). Therefore, 100 participants concluded the study; 40 of them were CDS+ADHD whereas 60 of them were solely diagnosed with ADHD (see Figure 1).

### Measures

All participants were evaluated through clinical interviews based on The Kiddie Schedule for Affective Disorders and Schizophrenia for School-Aged Children – Present and Lifetime Version (K-SADS-PL) and DSM-5 TR by child and adolescent psychiatrists. Also, assessment scales from different sources and settings were collected to provide consistency of diagnosis.

#### Sociodemographic Information Form

This form was created by the researchers to gather information about the participants and their caregivers, including their sociodemographic details.

#### Kiddie Schedule for Affective Disorders and Schizophrenia for School-Age Children – Present and Lifetime Version, DSM-5 November 2016-Turkish Adaptation

Kiddie Schedule for Affective Disorders and Schizophrenia for School-Age Children – Present and Lifetime Version DSM-5 (November 2016 version), which is revised from the previous one based on DSM-5 diagnostic criteria by Kaufman et al.^17^ It is a semi-structured diagnostic interview administered by a trained clinician to both caregivers and children to evaluate the current and past psychopathologies of children and adolescents.

#### Conners’ Teacher Rating Scale and Conners’ Parent Rating Scale

The 28-item Conners’ Teacher Rating Scale and 48-item Conners’ Parent Rating Scale are Likert-type scales consisting of 4 points filled out by teachers and parents respectively. They were developed to measure symptoms of ADHD and related disorders by Goyette et al.^18^ In this study, these scales were used to assess ADHD symptomatology.

#### Barkley Child Attention Scale

Barkley Child Attention Scale (BCAS)^11^, which is a 12-item 4-point Likert-type scale, was filled out by parents and used to evaluate CDS symptom severity in this study.

### Neuropsychological Tests

To evaluate the CF of the participants, 4 different NPTs that are listed as follows were used: Wisconsin Card Sorting Test (WCST), Stroop Color–Word Test (SCWT), Verbal Fluency Test (VFT) and Color Trails Test (CTT). These assessments have been extensively utilized in the literature to investigate CF.^19^A single specialist manually administered all the tests to ensure consistency and fairness in the performance evaluation. Each child was assessed individually in a quiet room during a 40-minute session; time-outs were given between tasks. Additionally, children who were taking ADHD medication were asked to skip their daily dose on the day of the test to ensure that the medication did not affect the test performance.

#### Wisconsin Card Sorting Test

Wisconsin Card Sorting Test evaluates the executive activities such as planning, organization, shifting of attention, and CF.^20^ This test involves matching a deck of cards to different lists by color, shape, or number without disclosing a specific rule. After a series of correct answers, the pattern is changed without warning, and the participant is expected to find the new rule. The manual that was applied in this study included 12 indexes. Among these, perseverative errors (PE; incorrect responses based on the previous pattern despite receiving negative feedback) and perseverative responses (PR; any repeated correct or incorrect response) are the most relevant for CF.^21^

#### Stroop Color–Word Test

The Stroop Color–Word Test assesses CF by utilizing the color–word interference effect. It includes 5 sections that record completion time across 3 stimulus conditions: (1) congruent stimuli, where the word and ink color match, assessed in sections 1 and 3; (2) incongruent stimuli, where the word meaning and ink color are in conflict, assessed in sections 2 and 5; and (3) neutral stimuli, consisting of either color or text presented without interference, assessed in section 4. Karakaş et al^22^emphasizes that section 5 is the most critical part of the test in terms of interference effect.

#### Verbal Fluency Test

Verbal Fluency Test consists of 2 subtests named Semantic Verbal Fluency (SVF) and Phonological Verbal Fluency (PVF) that measure a person’s verbal productivity. Semantic Verbal Fluency involves generating words from a predefined category within 60 seconds, and PVF participants are asked to produce words that begin with a predetermined letter, excluding proper nouns, within 60 seconds. It has been shown that executive functions such as CF, inhibition, and processing speed are the strongest determinants of VFT performance.^23^

#### Color Trails Test

Color Trails Test is a language-free variant of the Trail Making Test, which is an NPT that measures a person’s motor coordination, visual, and spatial abilities.^24^In this test, the participant is asked to move their fingers in a certain pattern and follow lines or dots. The test consists of 2 subtests; CTT1 assesses psychomotor speed and attention by connecting the dots in a sequential pattern, whereas CTT2 focuses on higher cognitive functions such as CF by connecting dots in an alternating and specific pattern.^25^

### Ethics Statement

The parents of the sample group were informed in detail about the study procedure and their written informed consent was obtained. This study was approved by the Ethics Committee of Erenköy Mental and Neurological Diseases Training and Research Hospital (Approval No.: 15; Date: March 7, 2022).

### Statistical Analysis

The data were analyzed using version 25.0 of SPSS (IBM SPSS Corp.; Armonk, NY, USA). First, the data were scrutinized for missing values and outliers. In the statistical analyses of the data, categorical variables were presented with case counts and frequency values, while continuous variables were reported using mean and SD values. The normality of data distribution was examined using the Kolmogorov-Smirnov test. Data that did not show a normal distribution were presented using the median and interquartile range. The chi-square test was used for comparing categorical variables. For comparisons of continuous variables between 2 groups, the independent samples t-test was used if normal distribution was satisfied, and the Mann–Whitney *U*-test was used when normality criteria were not satisfied. For continuous variables showing a normal distribution, Pearson correlation analysis was applied, whereas Spearman correlation analysis was used for continuous variables that did not follow a normal distribution. A significance level of *P* < .05 was accepted as statistically significant in the analyses.

## Results

This study examined a sample of 100 participants, divided into 2 groups: CDS+ADHD (n = 40) and ADHD-only (n = 60). Sociodemographic characteristics of the sample are shown in Table 1. The mean age of participants was similar across groups, and the prevalence of male gender was apparent. There were no significant differences in age or gender distribution between groups (*P* > .05).

Table 2 displays group differences for in each NPTs. Firstly, Wisconsin Card Sorting Test (WSCT) indexes are shown. The CDS+ADHD group made more total errors in the WCST compared to the ADHD-only group (t = −3.14, *P* = .003). Also, participants with CDS+ADHD had a fewer total number of correct responses (t = 4.37, *P* < .001) and categories completed (Z = −2.51, *P* = .012) than the ADHD-only group. The CDS+ADHD group showed a significantly greater score of PRs (Z = −2.38, *P* = .017), PEs (Z = −2.63, *P* = .009), non-PEs (Z = −2.36, *P* = .021), and PE percentage (Z = −3.81, *P* < .001) compared to the ADHD-only group. On the other hand, the CDS+ADHD group scored lower than the ADHD-only group in conceptual level responses (Z = −4.08, *P* < .001) and percentage of conceptual level responses (t = 2.90, *P* = .005) and failure to maintain set (Z = −2.62, *P* = .009). The comparison of SCWT and CTT subtests completion times in between groups did not show a statistically significant difference (*P* > .05). Finally, VFT scores of sample groups are shown in subtests. In terms of SVF_31-60 _(t = 2.26, *P* = .026) and SFT_Total_ (t = 2.17, *P* = .035) scores, a statistically significant difference was found between ADHD+CDS and ADHD-only groups; however, no statistically significant differences were found on the other scores of VFT (*P* > .05).

## Discussion

In this study, the aim was to investigate how CDS influences CF in children with ADHD. Existing literature provides strong evidence for the impact of ADHD on CF. For instance, a meta-analysis conducted by Townes et al^26^found that children and adolescents with ADHD exhibit significantly greater impairments in CF compared to their typically developing counterparts. Due to the limited number of studies on CDS’s effect on CF, the findings are controversial and in need of replication. However, more recent studies have revealed impaired CF,^9^^,^^27^^,^^28^ a finding that has been successfully replicated in this study. In this study, it was found that several indexes of the WCST and VFT significantly differ between ADHD-only and CDS+ADHD groups. Therefore, these results support the hypothesis that the CF of children with ADHD is further affected by CDS symptomology. To the authors’ knowledge, this is the first study that examines CDS’s effect on CF by using NPTs consisting of WCST, SCWT, VFT, and CTT simultaneously.

In WSCT, the CDS+ADHD group had a significantly higher total number of errors, completed fewer categories, and their total number of responses was lower than the ADHD-only group. Also, they had significantly more PEs and PRs compared to ADHD-only group. As shown in studies by Yates et al^29^ collectively these findings indicate that if CDS accompanies ADHD in a person, they tend to be more cognitively inflexible and display impaired performance. Moreover, non-PEs were found to be greater in the CDS+ADHD group which shows difficulty in forming a concept. Despite these findings in WSCT, no difference in completion time of SCWT sections was observed between the groups. Both the WCST and SCWT measure CF, yet they do so through distinct mechanisms. Stemme et al^30^explain this difference precisely by “stimulus congruency effect of set shifting tasks is studied using Stroop-like tests while uninstructed set shifts are examined by WCST-like tasks.” Therefore, the lack of difference even in section 5, which considered a key indicator for interference, can be explained from this perspective.

In terms of VFT, SFT_31-60_, and SFT_Total _results were found to be significantly higher in the ADHD-only group. This finding may be attributed to variation in cognitive demand throughout the test and motivation to achieve a result. Cognitive effort is likely to be higher in the second half of the task (SFT_31-60_) compared to the first half due to the increased requirement for sustained attention, working memory, and lexical retrieval in semantic memory.^31^ On the other hand, CDS symptomology is found to be associated with less willingness to work.^32^These 2 factors can explain the observed results.

No significant differences in CTT completion times were observed between groups, although the CDS+ADHD group took a longer time to complete both subtests. CTT1 mainly assesses processing speed and CDS’s effect on processing speed is contradictory. Thus, the current findings for the CTT1 subtest are compatible to conflicting nature of the literature. However, seeing that CTT2 is closely related to CF, the absence of significant differences warrants further analysis. This difference can be explained by the distinct methodology of NPTs, as these assessments evaluate CF through distinct executive functions.

Although the same level of dysfunction in CF could not be replicated in all the NPTs that were applied in this study, there are several possible reasons for this result. Firstly, NPTs can be used to assess CF; however, their scope is not solely limited to CF. This creates the problem of task impurity, which can be observed in all NPTs since they are not limited to a single task. Even if measuring CF with these NPTs were hypothesized, the other variables they measure could not be excluded. Secondly, investigating CF was undeniably a challenge due to its unique and hard-to-define construct, which explains the limited literature on this subject.^16^ CF is a higher-order construct that requires and includes several complex executive functions. The recommendations based on this limitation will be described at the end of the discussion. Lastly, Mayes et al^33^ conducted a study on the assessment of CDSs using NPTs and found a ground-breaking result, suggesting that existing NPTs cannot measure CDS traits with reliability. Given the discrepancy of the findings in this area, the development of new assessment tools may be necessary, drawing insights from the shortcomings of current literature.

The findings of this study can be adapted to clinical practice. Firstly, Wiggs et al^34^proposed that mindful awareness practices may be beneficial in cases of CDS+ADHD, though their findings are preliminary. As mindfulness practices are strongly connected to enhancement in CF,^35^ by deductive reasoning, it can be said that cognitive inflexibility may be a result of CDS. Therefore, the results of this study may shed light on the mechanism of these interventions. Secondly, NPTs that are used in this study are commonly preferred in clinical settings for a variety of purposes. Lower test scores than expected in ADHD-diagnosed children may be an indication for the screening of CDS and should be carefully evaluated.

This study has some limitations. First, it is designed as a single-centered cross-sectional study with a limited sample size, which means that the findings cannot be generalized to the entire population. Additionally, self-report tools were used for data collection, which may introduce bias by distorting causal relationships. For future studies, the use of both NPTs and CF self-report scales supported by noninvasive neuroimaging approaches for CF in CDS is recommended to minimize task impurity and develop a better understanding of the impaired components.

In conclusion, findings of this study revealed that ADHD cases accompanied by CDS can be suffering from CF dysfunctions compared to ADHD-only cases, yet these findings need to be replicated. The hope is that this research will pave the way for new studies using novel or different methods focusing on this field.

## Figures and Tables

**Table 1.** Sociodemographic Characterization of the Study Sample

		**CDS+ADHD** **(n = 40)**	**ADHD-Only** **(n = 60)**	**Statistical Analysis**	***P***
Age, M ± SD		9.97 ± 1.82	10.00 ± 1.72	*t* = 0.10	.920
Gender, n (%)	Male	27 (67.5)	48 (80.0)	*Χ*^2^ = 2.00	.157
	Female	13 (32.5)	12 (20.0)		

CDS, cognitive disengagement syndrome; M, mean; *t*, independent samples *t*-test; *X*^2^, chi-square test.

**Table 2.** Comparison of Neuropsychological Tests Between Groups

		**CDS+ADHD (n = 40)**	**ADHD-only (n = 60)**	**Statistical Analysis**	***P***
**WCST indexes**					
Total number of trials administered, M ± SD		118.03 ± 18.16	116.12 ± 15.27	*t* = −0.57	.572
Total number of errors, M ± SD		49.15 ± 23.42	36.00 ± 15.06	*t *= −3.14	.003*
Total number of correct responses, M ± SD		68.90 ± 14.44	80.20 ± 9.44	*t* = 4.37	<.001**
Categories completed, median, (IQR)^a^		4.00 (3.00-6.00)	6.00 (4.00-6.00)	Z = −2.51	.012*
Perseverative responses, median (IQR)^a^		28.50 (11.75-39.75)	22.00 (13.00-28.00)	Z = −2.38	.017*
Perseverative error, median (IQR)^a^		26.00 (11.50-34.75)	20.00 (11.25-25.75)	Z = −2.63	.009*
Non-perseverative errors, M ± SD		22.45 ± 13.02	16.88 ± 8.94	*t* = −2.36	.021*
Perseverative error percentage, median (IQR)^a^		22.66 (17.00-32.62)	16.75 (11.54-20.45)	Z = −3.81	<.001**
Number of trials to complete first category, median (IQR)^a^		12.00 (11.00-14.75)	11.00 (11.00-12.00)	Z = −1.68	.094
Conceptual level response, median (IQR)^a^		59.50 (42.25-66.00)	68.00 (61.25-76.75)	Z = −4.08	<.001**
Conceptual level response percent, M ± SD		47.87 ± 22.56	59.72 ± 15.43	*t* = 2.90	.005*
Failure to maintain set, median (IQR)^a^		0.50 (0.00-2.00)	2.00 (1.00-2.75)	Z = −2.62	.009*
**Completion time of SCWT sections (sec)**					
Section 1, median (IQR)^a^		11.50 (9.00-15.50)	11.50 (10.00-13.00)	Z = −0.33	.742
Section 2, M ± SD		13.28 ± 4.62	12.85 ± 4.30	*t* = −0.47	.639
Section 3, M ± SD		19.30 ± 7.43	18.20 ± 5.49	*t* = −0.85	.397
Section 4, median (IQR)^a^		26.00 (19.50-36.50)	25.00 (19.50-30.50)	Z = −0.82	.412
Section 5, M ± SD		44.43 ± 22.59	41.82 ± 16.41	*t* = −0.67	.505
**Completion time of CTT subtests (sec)**					
CTT1, median (IQR)^a^		37.50 (24.25-52.00)	29.50 (23.00-47.25)	Z = −1.26	.208
CTT2 median (IQR)^a^		84.50 (64.00-128.00)	78.50 (55.25-100.75)	Z = −1.52	.129
**Scores of VFT subtests**					
SFT, M ± SD	SFT_1-30_	9.95 ± 3.02	10.75 ± 3.43	*t* = 1.20	.234
	SFT_31-60_	3.40 ± 2.02	4.38 ± 2.20	*t* = 2.26	.026*
	SFT_Total_	13.35 ± 4.14	15.24 ± 4.43	*t* = 2.17	.035*
PFT_Total_, M ± SD		17.08 ± 8.46	20.60 ± 9.90	*t* = 1.85	.068

Interquartile range is presented as the difference between third and first quartiles.

CDS, cognitive disengagement syndrome; CTT, Color Trail Test; IQR, interquartile range; M, mean; PFT_Total_, Total score of Phonemic Fluency Test; SCWT, Stroop Color–Word Test; sec, seconds;SFT, Semantic Fluency Test; SFT_1-30_, SVF score in first 30 seconds; SFT_31-60_, SVF score between 31 and 60 seconds; SFT_Total_, SVF score in 1 minute; *t*, independent samples *t*-test; VFT, Verbal Fluency Test; WCST, Wisconsin Card Sorting Test; Z, Mann–Whitney *U*-Test.

^a^Data median.

*P < .05.

**P < .001.

## Data Availability

The data that support the findings of this study are available on request from the corresponding author.
